# The incidence and risk factors of perioperative cardiac complications in noncardiac major surgery in high-altitude areas: A prospective trial in Tibet autonomous region, China

**DOI:** 10.3389/fcvm.2023.1158711

**Published:** 2023-04-03

**Authors:** Qianmei Zhu, Hanyu Duan, Zijia Liu, Yi Li, Yuelun Zhang, Labaciren, Le Shen, Yuguang Huang

**Affiliations:** ^1^ Department of Anesthesiology, Peking Union Medical College Hospital, Chinese Academy of Medical Sciences and Peking Union Medical College, Beijing, China; ^2^ Department of Anesthesiology, Tibet Autonomous Region People’s Hospital, Lhasa, China; ^3^ Medical Research Center, Peking Union Medical College Hospital, Chinese Academy of Medical Sciences and Peking Union Medical College, Beijing, China

**Keywords:** Tibet autonomous region, high altitude, perioperative cardiac complications, noncardiac surgery, risk factor

## Abstract

**Background:**

The risk of perioperative cardiac complications (PCCs) in patients living in high-altitude areas may increase with more adverse clinical outcomes due to the special geographical environment, which has not yet been studied. We aimed to determine the incidence and analyze risk factors for PCCs in adult patients undergoing major noncardiac surgery in the Tibet Autonomous Region.

**Methods:**

This prospective cohort study enrolled resident patients from high-altitude areas receiving major noncardiac surgery in Tibet Autonomous Region People's Hospital in China. Perioperative clinical data were collected, and the patients were followed up until 30 days after surgery. The primary outcome was PCCs during the operation and within 30 days after the surgery. Logistic regression was used to build the prediction models for PCCs. A receiver operating characteristic (ROC) curve was used to evaluate the discrimination. A prognostic nomogram was constructed to generate a numerical probability of PCCs for patients undergoing noncardiac surgery in high-altitude areas.

**Results:**

Among the 196 patients living in high-altitude areas involved in this study, 33 (16.8%) suffered PCCs perioperatively and within 30 days after surgery. Eight clinical factors were identified in the prediction model, including older age (*P* = 0.028), extremely high altitude above 4,000 m (*P* = 0.442), preoperative metabolic equivalent (MET) < 4 (*P* = 0.153), history of angina within 6 months (*P* = 0.037), history of great vascular disease (*P* = 0.073), increased preoperative high sensitivity C-reactive protein (hs-CRP) (*P* = 0.072), intraoperative hypoxemia (*P* = 0.025) and operation time >3 h (*P* = 0.043). The area under the curve (AUC) was 0.766 (95% confidence interval: 0.785–0.697). The score calculated from the prognostic nomogram predicted the risk of PCCs in high-altitude areas.

**Conclusion:**

The incidence of PCCs in resident patients living in high-altitude areas who underwent noncardiac surgery was high, and the risk factors included older age, high altitude above 4,000 m, preoperative MET < 4, history of angina within 6 months, history of great vascular disease, increased preoperative hs-CRP, intraoperative hypoxemia, and operation time >3 h. The prognostic nomogram of this study could help to assess the PCCs for patients in high-attitude areas undergoing noncardiac surgery.

**Clinical Trial Registration:**

ClinicalTrials.gov ID: NCT04819698.

## Introduction

Cardiac complications are uncommon but are reported as the primary cause of postoperative death (1–3). Older age, history of cardiovascular disease, peripheral and major vascular disease, cerebral infarction, renal insufficiency, and diabetes have been recommended as preoperative risk comorbidities for patients experiencing perioperative cardiac complications (PCCs) (2, 4, 5).

The hypoxic environment in high-altitude areas leads to a decrease in arterial partial pressure of oxygen (PaO_2_) and pulse oxygen saturation (SpO_2_). Long-term exposure to chronic hypoxia can cause pulmonary arterioles to contract, which leads to pulmonary artery hypertension (PAH) and increased right ventricular load (6, 7). In addition to cardiac overload, the incidences of hyperhemoglobinemia, hyperlipemia, and hypercoagulability are also higher than those found in patients living in plain areas (8). Furthermore, reduced oxygen supply and hyperlipidemia contribute to coronary heart disease and other cardiac events (9). Living at high altitude also increases the risks of deep vein thrombosis and pulmonary embolism after surgery, which is believed to be related to the pathological state of high-altitude polycythemia, venous stasis and hypercoagulability (10–13). Therefore, these patients may have an increased risk of adverse cardiac events during the perioperative period. However, few studies have elaborated the incidence and risk factors for limited case resources in high-attitude areas thus far.

The Tibet Autonomous Region of China is known as the “roof of the world”, with an average altitude of more than 4,000 m. In this study, we prospectively explored the incidence of PCCs for patients in the Tibet Autonomous Region undergoing noncardiac surgery and analyzed the risk factors. This study will help optimize perioperative care management and enhance the prognosis of patients in high-altitude areas.

## Materials and methods

### Study population

This study is a prospective cohort study of adult patients living in high-altitude areas receiving major noncardiac surgeries in Tibet Autonomous Region People's Hospital, China, between June 2021 and August 2022. The altitude of Tibet Autonomous Region People's Hospital is 3,568 m. The study was approved by the Medical Ethics Committee of the People's Hospital of Tibet Autonomous Region (Ethics No: ME-TBHP-20-26) and was performed in accordance with the Declaration of Helsinki. Written informed consent was obtained from all patients.

All patients who met the following criteria and voluntarily participated in this study were considered for inclusion consecutively, and the follow-up contents and methods were kept the same. The inclusion criteria were as follows: patients received general anesthesia for intermediate- to high-risk noncardiac surgery; lived in high-altitude areas above 3,000 m for more than 5 years; and were ≥50 years old. The types of surgery were considered based on the American College of Cardiology (ACC)/American Heart Association (AHA) guidelines of perioperative cardiovascular evaluation (14, 15), including abdominal and retroperitoneal surgery (e.g., included laparoscopic surgery), vascular surgery (e.g., included large vessels and peripheral vessels), orthopedic surgery, neurosurgery, head and neck surgery, and thoracic surgery. Exclusion criteria included patient refusal, emergency operation, low-risk surgery, operation under local anesthesia, cardiac surgery, and American Society of Anesthesiologists (ASA) classification V.

### Preoperative evaluation and data collection

The general information of patients was prospectively collected, including sex, age, nationality, smoking history, altitude of habitation, previous medical history, history of peripheral vascular disease, cerebrovascular disease, cardiovascular disease, hypertension and treatment, diabetes and treatment, hyperthyroidism, chronic obstructive pulmonary disease (COPD) and asthma. Hypertension (HTN) with irregular treatment in this study means that patients did not regularly take the antihypertensive drugs prescribed by the doctor or never took any antihypertensive drugs after the diagnosis of hypertension. The metabolic equivalent (MET), New York Heart Association (NYHA), and ASA classification were evaluated and recorded.

All patients completed routine preoperative physical examinations, including body mass index (BMI), preoperative heart rate (HR), blood pressure (BP), and SpO_2_. The results of preoperative laboratory examination, such as hemoglobin level and coagulation index, 12-lead electrocardiogram (ECG), and transthoracic echocardiography, were noted. Preoperative pulmonary hypertension (PAH) was diagnosed as pulmonary artery pressure >35 mmHg by transthoracic echocardiography (16, 17).

### Procedures

No preoperative medication was administered. After entering the operation room, SpO_2_, BP, HR, 3-lead ECG, and end tidal carbon dioxide (EtCO_2_) were continuously monitored. Surgical procedures were performed under general anesthesia with total intravenous anesthesia (TIVA) or sevoflurane inhalation. The bispectral index (BIS) was kept between 40 and 60. A lung protective ventilation strategy was performed during the operation with a tidal volume of 6 to 8 ml/kg of ideal body weight, titrimetric positive end expiratory pressure (PEEP) and recruitment maneuver. Vasopressors, fluids, or a combination of both were used if systolic arterial pressure decreased above 20% of the base value. Monitoring of patients' temperature was mandatory to avoid hypothermia and hyperthermia. Maintenance of normothermia was implemented perioperatively through active warming devices. Perioperative multimodal analgesia and postoperative nausea and vomiting prevention were performed. The total volume of fluid infusion, blood loss, blood transfusion, urine volume, operation time, operative site, and anesthesia protocol were all recorded. Hypotension was defined as systolic arterial pressure below 80% of the baseline. Prolonged intraoperative hypoxemia was defined as PaO_2_ < 60 mmHg (8.00 kPa) or SpO_2_ < 90% over 10 min.

HR, BP, SpO_2_, and 3-lead ECG monitoring were carried out for at least 24 h after surgery. If there were any typical or atypical cardiac symptoms, the patients received echocardiography and enzymography of the myocardium immediately. Patients were followed up 30 days after the surgery.

### Outcomes

The primary outcome was the occurrence of PCCs during the operation and within 30 days after the surgery. PCCs were defined as acute coronary syndrome (ACS), heart failure (HF), new-onset severe arrhythmia, nonfatal cardiac arrest, and cardiac death (15, 18–22). ACS included ST-elevation myocardial infarction (STEMI) and non-ST-elevation acute coronary syndrome (NSTE-ACS). NSTE-ACS was further subdivided into non-ST-elevation MI and unstable angina. New-onset severe arrhythmia included malignant arrhythmia, severe sinus bradycardia, paroxysmal supraventricular tachycardia (PSVT), atrial fibrillation (AF), frequent ventricular premature contractions (VPCs), and severe atrioventricular block (AVB) that needed to be treated with drugs or electrical conversion. The definitions of PCCs and adjudication for confirmation are described in detail in Supplementary Table S1. Myocardial enzymes were detected if necessary to diagnose myocardial injury after noncardiac surgery (MINS). MINS is usually recognized when the concentration of cardiac troponin (cTn) exceeds the 99th percentile value of the upper limit of the reference range of the normal population, which was defined in this study as a peak cTnI level > 0.06 µg/L. The type of PCCs and the exact occurrence time were recorded if any of these complications were present from admission to the operating theatre to 30 days after the surgery. If successive PCCs of more than one type occurred, the first cardiac complication was recorded.

### Statistical analysis

Categorical variables were presented as numbers and percentages (%), and continuous variables were presented as the mean ± standard deviation (SD) if the data distribution was normal or else presented as the median and interquartile range (IQR). First, the recorded preoperative and intraoperative factors were analyzed by univariate analysis, such as the chi-square test, *t test*, or Mann‒Whitney *U test*, according to different data distributions. Factors with significant differences (*P* < 0.1) and factors with clinical significance based on previous literature or clinical experience were all included in logistic regression analysis to screen out independent risk factors for PCCs. The receiver operating characteristic (ROC) curve was used to evaluate the discrimination of the indicators to predict PCCs. The area under the curve (AUC) was also calculated (23). Calibration was evaluated using Hosmer and Lemeshow's goodness of fit test and calibration plot. A two-sided *P* value < 0.05 was considered statistically significant except for the Hosmer and Lemeshow goodness of fit test, in which a two-sided *P* value < 0.1 was considered significant. A prognostic nomogram was constructed to generate a numerical probability of PCCs at high attitude. Statistical analysis was performed using R (Austria; version 3.5.2) with the “pROC” and “rms” packages (24, 25).

## Results

### Demographics

**Figure 1:**
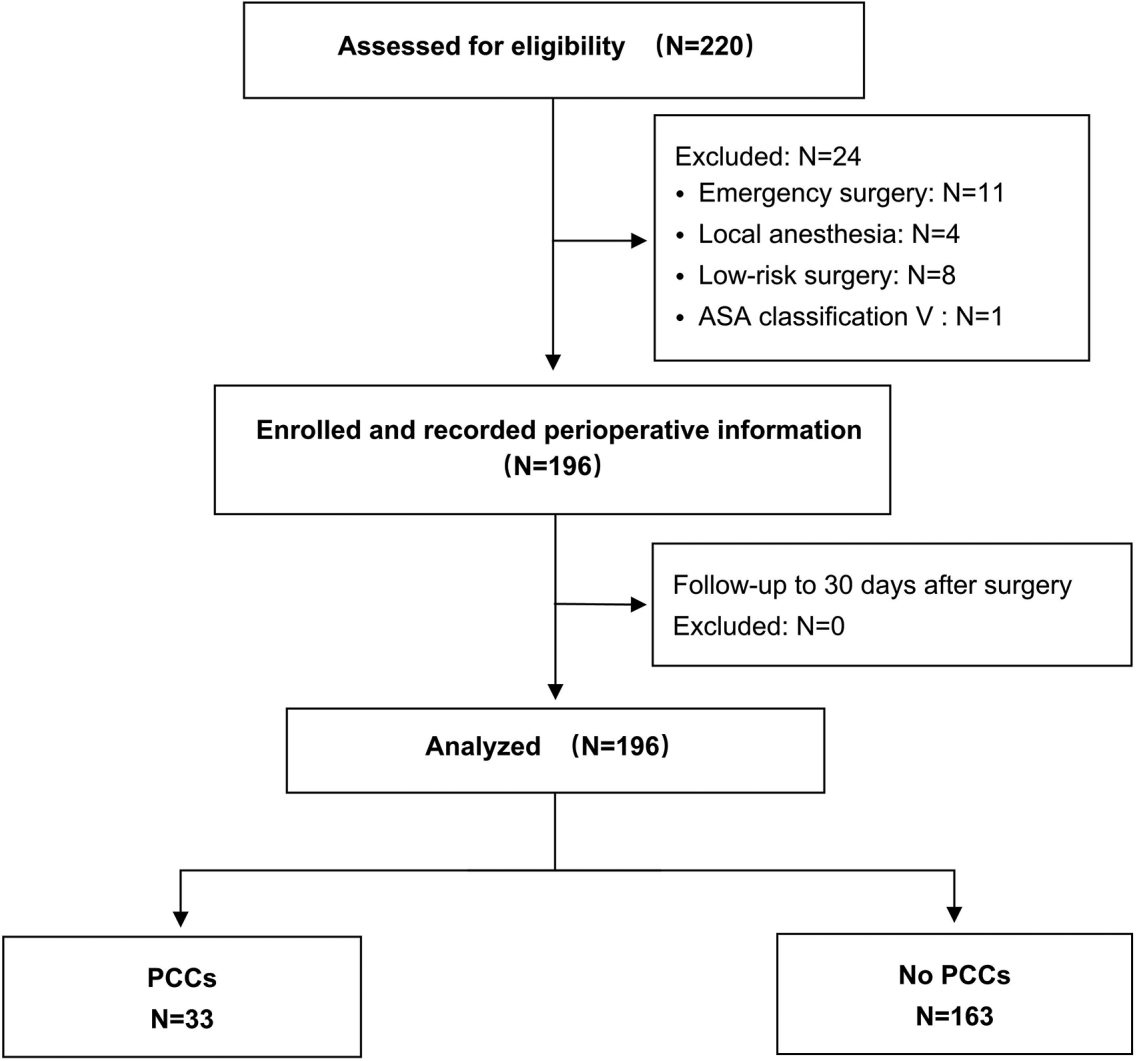
Flow chart of patient enrollment and analysis. PCCs, perioperative cardiac complications.

In this study, a total of 220 consecutive patients were assessed for eligibility from June 1st, 2021, to August 31st, 2022, of whom 24 were excluded before surgery because they were judged ineligible. In total, 196 patients were enrolled, underwent surgery and were followed up until 30 days after surgery (Figure 1). Among these patients, the median age was 60.4 ± 7.8 years. Most of them were Tibetans (94.4%), the majority of whom (90.3%) had lived in high-altitude areas above 3,500 m for a long time. The demographic characteristics and types of surgery are summarized in Table 1.

**Table 1:** Demographics and surgery types of the study population (*N* = 196).

Characteristics	Mean ± SD, or number (%)
Age (years)	60.4 ± 7.8
Male	89 (45.4)
**Nationality**	
Tibetan	185 (94.4)
The han nationality	9 (4.6)
The hui nationality	2 (1.0)
BMI (kg/m^2^)	25.0 ± 4.5
Smoking history	41 (20.9)
Current smoker	20 (10.2)
Exercise capability < 4MET	134 (68.4)
**Altitude of the patient’ residence**	
<3,500 m	19 (9.7)
3,500 m ≤ residence < 4,000 m	94 (48.0)
4,000 m ≤ residence < 4,500 m	73 (37.2)
4,500 m ≤ residence < 5,000 m	8 (4.1)
Residence ≥ 5000 m	2 (1.0)
**Comorbidities**	
MI	5 (2.6)
HF	7 (3.6)
HTN	93 (47.4)
HTN with irregular treatment	67 (34.2)
Arrhythmia	43 (21.9)
Main and peripheral vascular disease	50 (25.5)
Cerebral infarction	8 (4.1)
Cerebral hemorrhage	4 (2.0)
Diabetes	12 (6.1)
COPD	75 (38.3)
Asthma	9 (4.6)
**NYHA classification**	
I	102 (50.2)
II	89 (45.4)
III	5 (2.6)
**ASA status**	
II	118 (60.2)
III	76 (38.8)
IV	2 (1.0)
**Types of surgery**	
Main and peripheral vascular procedures	9 (4.6)
Thoracic surgeries	20 (10.2)
Abdominal surgeries	88 (44.9)
Orthopedic surgeries	33 (16.8)
Neurology surgery	18 (9.2)
Head and neck surgeries	22 (11.2)
Other types of surgery	6 (3.1)

Results are presented as the mean (SD) or n (%). Other types of surgery included retroperitoneal surgery, transurethral prostate resection and vaginal hysterectomy.

ASA, American Society of Anesthesiologists; BMI, body mass index (weight/height^2^); COPD, chronic obstructive pulmonary disease; HF, heart failure; HTN, hypertension; MET, metabolic equivalent; MI, myocardial infarction; NYHA, New York Heart Association; SD, standard deviation.

### Incidence of PCCs

A total of 33 patients (16.8%) had cardiac complications within 30 days after surgery (14 patients intraoperatively, 14 patients postoperatively, and 5 patients experienced complications both intraoperatively and postoperatively). Among the 14 patients with postoperative cardiac complications, 8 patients (57.1%) had PCCs within the first 3 days after surgery. The details of PCCs incidence are shown in Table 2.

**Table 2:** Detailed information on perioperative cardiac complications.

PCCs component outcomes	*N*	Proportion in PCCs (%)	Cumulative incidence in the whole population (%)
Unstable angina	7	21.2	3.6
New-onset severe arrhythmia	25	75.8	12.8
Sinus bradycardia	10	30.3	5.1
PSVT	4	12.1	2.0
AF	1	3.0	0.5
Frequent VPC	6	18.2	3.1
AVB	4	12.1	2.0
Nonfatal cardiac arrest	1	3.0	0.5
Total PCCs	33	100.0	16.8

AF, atrial fibrillation; AVB, atrioventricular block; PCCs, perioperative cardiac complications; PSTV, paroxysmal supraventricular tachycardia; PVC, ventricular premature contractions.

Arrhythmia (*n* = 25, 75.8%) was the most common PCCs in this study, including sinus bradycardia, PSVT, AF, frequent VPC and AVB. Among these 25 patients, 22 patients had cardiac enzyme detection after the operation, and 2 patients with PSVT (2/4) and 1 patient with AF (1/1) were in line with MINS. Seven patients experienced unstable angina during the perioperative period, of whom 5 patients underwent myocardial enzyme examination and were diagnosed with MINS. No patient had perioperative heart failure. One patient suffered nonfatal cardiac arrest, accompanied by elevated myocardial enzymes. One patient with PCCs (intraoperative bradycardia) died 10 days after the operation due to postoperative stroke. Among 163 patients without PCCs, a total of 29 patients were tested for myocardial enzymes, all within the normal range of the level of cTnI.

### Independent risk factors of PCCs and ROC analysis

**Figure 2:**
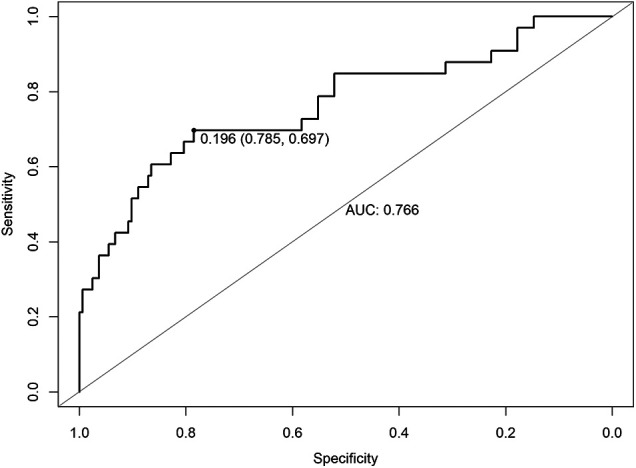
ROC of risk factors for PCCs for patients undergoing major noncardiac surgery in high-attitude areas. ROC, receiver operating characteristic; AUC, area under the curve.

Table 3 shows 14 possible risk factors for PCCs (*P* < 0.1) in univariate analysis. In addition, we added 4 other clinically important risk factors that may be related to the outcome, namely, preoperative MET < 4, preoperative ECG ST-T abnormality, intraoperative hypotension, and intraoperative transfusion. After analysis by logistic regression, 8 clinical factors were identified in the prediction model, including older age (*P* = 0.028), extremely high altitude above 4000 m (*P* = 0.442), preoperative MET < 4 (*P* = 0.153), history of angina within 6 months (*P* = 0.037), history of great vascular disease (*P* = 0.073), increased preoperative high sensitivity C-reactive protein (hs-CRP) (*P* = 0.072), intraoperative hypoxemia (*P* = 0.025) and operation time >3 h (*P* = 0.043) (Table 4). The AUC of the ROC analysis was 0.766 (95% CI: 0.785–0.697, *P* = 0.196) (Figure 2).

**Table 3:** Possible risk factors for PCCs in univariate analysis.

Variables	Patients with PCCs (*n* = 33)	Patients without PCCs (*n* = 163)	*χ^2^*/*t*/*Z*	*P* value
Age (years)	63.9 ± 8.8	59.7 ± 7.5	2.877	0.004
Attitude of residence >4,000 m	18 (54.5)	65 (39.9)	2.419	0.087
Preoperative MET < 4	8 (24.2)	54 (33.1)	1.002	0.215
History of MI	3 (9.1)	2 (1.2)	6.827	0.034
History of angina within 6 months	9 (27.3)	13 (8.0)	10.256	0.004
History of HF	3 (9.1)	4 (2.5)	3.510	0.094
History of HTN with irregular treatment	20 (60.6)	73 (44.8)	2.755	0.071
History of great vascular disease	14 (42.4)	36 (22.1)	5.974	0.015
Preoperative hs-CRP (mg/L)	14.79 (6.75,23.6)	6.32 (3.58,13.66)	−2.902	0.004
Preoperative ECG arrhythmia	24 (72.7)	88 (54.0)	3.935	0.035
Preoperative ECG ST-T abnormality	18 (54.5)	68 (41.7)	1.834	0.123
Preoperative PAH	7 (21.2)	10 (6.1)	7.876	0.011
Intraoperative hypotension	15 (45.4)	67 (41.1)	0.213	0.392
Intraoperative hypoxemia	10 (30.3)	14 (8.6)	12.042	0.002
Intraoperative transfusion	7 (21.2)	28 (17.2)	0.304	0.369
Intraoperative bleeding (ml)	200 (80,600)	100 (45,300)	−2.286	0.022
Operation time >3 h	17 (51.5)	55 (33.7)	3.730	0.043
ASA classification	2 (2,3)	3 (2,3)	12.655	0.002

The data with a normal distribution were compared by group *t* test; the data with a nonnormal distribution between the two groups were compared by Mann–Whitney *U* test, and the counting data were expressed by chi-square test.

PCCs, perioperative cardiac complications; MET, metabolic equivalent; MI, myocardial infarction; HF, heart failure; HTN, hypertension; hs-CRP, high sensitivity C-reactive protein; ECG, electrocardiogram; PAH, pulmonary artery hypertension; ASA, American Society of Anesthesiologists.

**Table 4:** Clinical factors included in the PCCs prediction model.

Variables	OR	95%CI	*P* value
Age (per year increase)	1.063	1.007–1.125	0.028
Attitude of residence >4,000 m	2.447	1.036–6.007	0.442
Preoperative MET < 4	2.187	0.736–6.443	0.153
History of angina within 6 months	3.286	1.047–10.058	0.037
History of great vascular disease	2.295	0.915–5.712	0.073
Preoperative hs-CRP (per mg/L increase)	1.013	0.998–1.029	0.072
Intraoperative hypoxemia	3.336	1.134–9.573	0.025
Time of operation >3 h	2.456	1.032–6.012	0.043

OR, odds ratio; CI, confidence interval; PCCs, perioperative cardiac complications; MET, metabolic equivalent; hs-CRP, high sensitivity C-reactive protein.

### Prognostic nomogram

**Figure 3:**
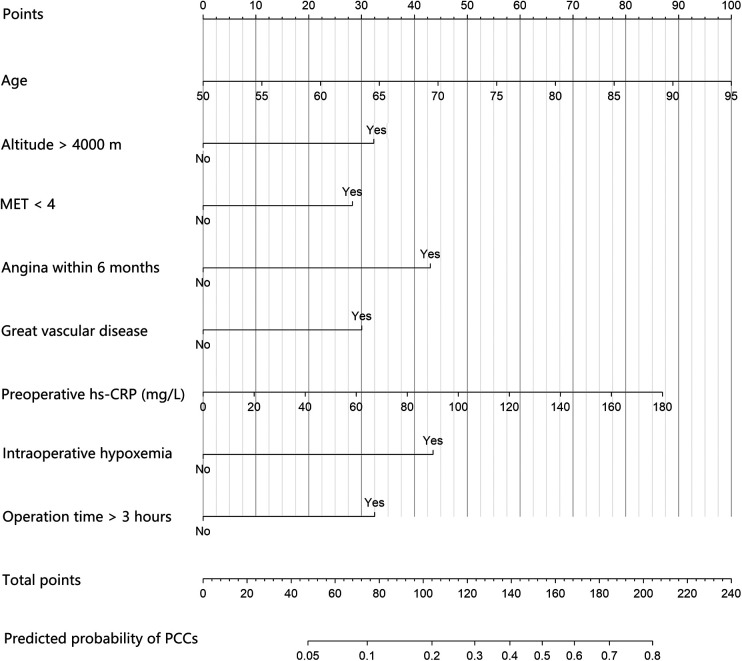
The prognostic nomogram of PCCs for patients undergoing major noncardiac surgery in high-attitude areas. An individual's value is located on each variable axis, and a line is drawn upward to determine the points received for each variable. Corresponding points for each clinical factor are as follows: 50 to 95 years old correspond to 0 to 100 points, respectively; 33 points for high altitude and 0 points for not; 28 points for preoperative MET < 4 and 0 points for not; 43 points for history of angina within 6 months and 0 points for not; 30 points for history of great vascular disease and 0 points for not; 0 to 180 mg/L preoperative hs-CRP correspond to 0 to 87 points, respectively; 44 points for intraoperative hypoxemia and 0 points for not; 33 points for operation time >3 h and 0 points for not. The sum of these points is located on the total point axis, and a line is drawn downward to the survival axes to determine the likelihood of PCCs. PCCs, perioperative cardiac complications; MET, metabolic equivalent; hs-CRP: high sensitivity C-reactive protein.

A prognostic nomogram integrated into the model was constructed (Figure 3). The total score was calculated according to the corresponding score assigned to each variable. The total score could correspond to the risk of PCCs in each patient. The nomogram had a concordance index of 0.766 and was well calibrated (Supplementary Figure S1).

## Discussion

The incidence of PCCs explored in this study was 16.8% in patients who had been living in high-altitude areas for a long time and underwent noncardiac surgery. The prediction model consisting of 8 perioperative risk factors had high discrimination and accuracy in predicting PCCs, which were older age, high altitude above 4,000 m, preoperative MET < 4, history of angina within 6 months, history of great vascular disease, increased preoperative hs-CRP, intraoperative hypoxemia, and operation time >3 h.

As previously reported, the incidence of PCCs in patients undergoing noncardiac surgery varies greatly from 0.9% to 8.6% according to different study populations (26–28). However, there is limited evidence showing the incidence of PCCs in high-altitude areas. The present study found that the incidence of PCCs in patients undergoing noncardiac surgery in Tibet was 16.8%, much higher than that in plain areas. Extremely high altitudes above 4,000 m were an independent risk factor for PCCs among the population in this study. Surely, the hypoxic environment at high altitude leads to a series of alterations in the metabolism and physiological functions of people living there, such as hypertension, pulmonary arterial hypertension (PAH), right heart failure, hyperlipidemia, elevated blood viscosity and erythrocytosis but relatively poor oxygen carrying capacity (29–31). Pulmonary artery pressure and the afterload of the heart may further increase during perioperative stress in low-oxygen environments, aggravating the imbalance of myocardial oxygen supply and demand, which is the most direct cause of perioperative arrhythmia and ischemic heart events.

In this study, arrhythmia was the main type of PCCs in plateau areas. Sinus bradycardia already existed in many patients at high altitude before operation, and the incidence of newly emerging sinus bradycardia during the perioperative period was also the highest among arrhythmias. In fact, this is the cardioprotective mechanism of the body under a high-altitude hypoxic environment (32). In addition, intermittent hypoxic conditioning has been shown to reduce myocardial reperfusion injury, myocardial infarction and tachyarrhythmia through *β*-adrenergic, *δ*-dopaminergic, and reactive oxygen-nitrogen signaling pathways (33). Therefore, during the perioperative period, bradycardia does not require urgent drug treatment. ACS is less common than arrhythmia in patients living at high attitude (34). Hypoxia is one of the main causes of myocardial ischemia during surgery (35). In addition, we found that more than half of postoperative cardiac complications occurred within the first 3 days after surgery, in agreement with the results in our previous study (15). Therefore, the first 72 h after operation is also a dangerous period for patients in high-altitude areas to have adverse cardiac events.

MET < 4 has been taken as a threshold for poor functional capacity and is associated with a high risk of adverse cardiac events after major noncardiac surgery (36, 37). In this study, we found that 68.4% of patients had preoperative MET < 4, and these patients were more prone to PCCs. However, it has been reported that only 56% of patients receiving noncardiac surgery in plain areas showed poor exercise tolerance in a prospective study (38). The preoperative activity tolerance of patients at high altitude is generally poor, so preoperative assessment of functional capacity is particularly important for patients at high altitude, and it is a simple but dynamic evaluation reflecting cardiopulmonary and systemic functions to assist static evaluations, such as computed tomography (CT) and echocardiography. Such a simple preoperative evaluation is also suitable for plateau areas where medical resources are relatively scarce.

hs-CRP is a nonspecific marker of acute systemic inflammatory reactions synthesized by the liver and one of the most powerful predictors of cardiovascular and cerebrovascular event risk (39, 40). It has been reported that an increase in preoperative hs-CRP levels is significantly related to the risk of adverse events after surgery (41), which is consistent with the results of our study on the plateau. Hypoxia can directly increase the hepatic synthesis of hs-CRP to promote the inflammatory response (42), indicating the more important predictive value of perioperative levels of hs-CRP in high-altitude localities.

In this study, 24 patients (12.2%) had intraoperative hypoxia, which was significantly associated with PCCs. Patients living in high-altitude areas for a long time have chronic hypoxia and may develop emphysema or cor pulmonale before surgery. Such patients are more likely to suffer from hypoxia under surgical stress than those in plain areas intraoperatively. A cohort study evaluating the relationship between the degree of hypoxemia and cardiac metabolic risk in permanent residents at high altitude found that every 5% decrease in resting SpO_2_ doubled the cardiac metabolic risk (43). These patients should thus be monitored and managed carefully during the perioperative period.

In addition, older age, history of angina within 6 months and severe vascular disease were also detected as risk factors in predicting PCCs in patients receiving noncardiac surgery at high altitude, which was consistent with previous research in the general population (5, 15, 44–46). Although these factors are uncontrollable, they have great predictive value for preoperative evaluation. A preoperative understanding of these patient factors is also important for the prevention of PCCs.

It has become an international consensus that postoperative cTn monitoring should be carried out for high-risk patients undergoing noncardiac surgery, and MINS is effective in predicting serious cardiac complications and mortality (1, 47). However, it seemed less significant and slightly difficult to achieve in plateau areas. We found that the most types of cardiac events after operation in plateau areas were arrhythmia instead of myocardial ischemia. The myocardial enzymes of patients with arrhythmia could be normal. Besides, timely postoperative cTnI measurement for all enrolled patients was difficult due to limited capacity of the laboratory. Therefore, our main criteria for determining perioperative PCCs in plateau areas in this study were mainly based on more accessible clinical symptoms, monitoring data and ECG, though cTnI monitoring for high-risk patients is also critical when available.

Our nomogram provides surgeons and anesthesiologists with the prediction of PCCs in patients receiving noncardiac surgery at high altitude. Therefore, the preoperative risk assessment can be made very quickly by calculating the 8 demographic and perioperative factors included. Through this nomogram, we can optimize perioperative management by improving preoperative oxygen reserve to fully ensure intraoperative oxygen supply and reduce long-term intraoperative SpO_2_ < 90% and by improving functional status and activity tolerance. Minimally invasive surgery should be adopted, and the operation time should be controlled as much as possible. For high-risk patients, the operation should be postponed or canceled until the patient's body is adjusted to a better condition.

There are several limitations in our study. Although the discrimination and accuracy are relatively high in predicting PCCs in those patients, we have not verified it internally or externally. The main reason is that the number of cases collected is relatively small; thus, a larger sample size needs to be studied in the future. In addition, due to the limited hospital diagnosis and treatment conditions in plateau areas, some data on possible risk factors could not be collected, such as myocardial enzymes and markers of myocardial injury closely related to PCCs. We acknowledge that without taking MINS as the main endpoint, the design scheme of this study may lack rigor, and the generalization of the research conclusion will be affected. Therefore, we still recommended that myocardial enzymes should be routinely monitored after surgery for high-risk patients, if conditions permit.

## Conclusion

In this study, we found that the incidence of PCCs in patients who had been living in the Tibet Autonomous Region for a long time and underwent noncardiac surgery was high. The prediction model that included the following 8 perioperative clinical factors had high discrimination and accuracy in predicting PCCs in high-altitude patients: older age, high altitude above 4,000 m, preoperative MET < 4, history of angina within 6 months, history of great vascular disease, increased preoperative hs-CRP, intraoperative hypoxemia, and operation time >3 h. Perioperative evaluation and management should be strengthened for surgical patients in high-altitude areas to reduce the risk of cardiac events. Future trials should address a larger sample size to verify the promotion value of this prediction model.

## Data Availability

The raw data supporting the conclusions of this article will be made available by the authors, without undue reservation.
