# Supplementary figures and images for: The incidence and risk factors of perioperative cardiac complications in noncardiac major surgery in high-altitude areas: A prospective trial in Tibet autonomous region, China
Source: Front Cardiovasc Med. 2023 Apr 3;10:1158711. doi: 10.3389/fcvm.2023.1158711 (PMC10106712; doi:10.3389/fcvm.2023.1158711)

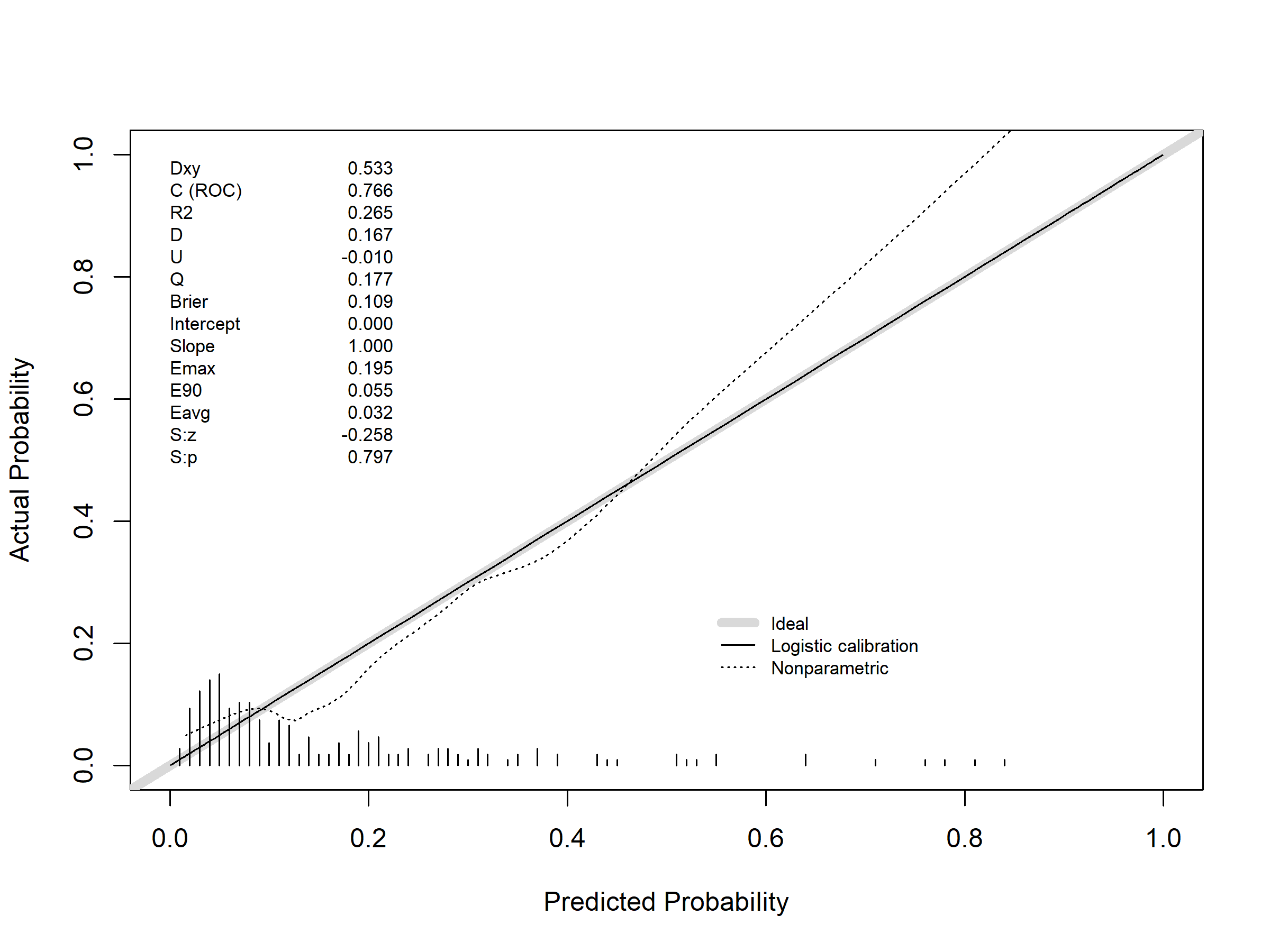

Supplement: Supplementary file 1 [file Image1.tiff]
