# Supplementary material for: The incidence and risk factors of perioperative cardiac complications in noncardiac major surgery in high-altitude areas: A prospective trial in Tibet autonomous region, China
Source: Front Cardiovasc Med. 2023 Apr 3;10:1158711. doi: 10.3389/fcvm.2023.1158711 (PMC10106712; doi:10.3389/fcvm.2023.1158711)
Supplement: Supplementary file 2 [file Table1.docx]

**Supplement Table S1** Definitions and confirmation of perioperative cardiac complications.

| **PCCs** | **Definition** | **Confirmation** |
| --- | --- | --- |
| **ACS** |  |  |
| **STEMI** | MI requires a cTnI levels rise above the 99th percentile, accompanied by chest pain, ST-segment changes or new-onset left bundle branch block, ventricular wall motion abnormalities, or angiography confirmation. | cTnI levels + clinical symptoms /ECG/ transthoracic echocardiography / angiography |
| **NSTE-ACS** | NSTE-ACS could be further subdivided into non-ST-elevation MI and unstable angina according to the cardiac biomarkers. |  |
| **HF** | Active clinical symptoms or physical examination findings of dyspnea, orthopnea, peripheral edema, jugular venous distention, rales, third heart sound, or chest X-ray with pulmonary vascular redistribution or pulmonary edema. | clinical symptoms+ physical examination +X-ray |
| **New-onset severe arrhythmia** | Electrocardiogram changes needing to be treated with drug or electrical conversion, including:   1. malignant arrhythmia (ventricular tachycardia or ventricular fibrillation) 2. severe sinus bradycardia (HR < 50 bpm and requires medication) 3. PSVT 4. atrial fibrillation and atrial flutter 5. frequent VPC needing drug therapy 6. II or III type atrioventricular block | ECG |
| **Cardiac arrest** | Loss of circulation prompting resuscitation requiring chest compressions, defibrillation, or both. | clinical symptoms + ECG |
| **Cardiac death** | Death with known fatal cardiac conditions, autopsy identified cardiac or vascular anomaly or any death with no obvious extracardiac cause could be established. | - |

**Abbreviations:** PCCs, perioperative cardiac complications; ACS, acute coronary syndrome; STEMI, ST-elevation myocardial infarction; NSTE-ACS, non-ST-elevation acute coronary syndrome; MI, myocardial infarction; HF, heart failure; HR, heart rate; PSVT, paroxysmal supraventricular tachycardia; VPC, ventricular premature contractions.
